# NR2B receptor blockade inhibits pain-related sensitization of amygdala neurons

**DOI:** 10.1186/1744-8069-5-21

**Published:** 2009-04-28

**Authors:** Guangchen Ji, Csilla Horváth, Volker Neugebauer

**Affiliations:** 1 Department of Neuroscience and Cell Biology, The University of Texas Medical Branch, Galveston, Texas 77555-1069, USA; 2 Pharmacological and Drug Safety Research, Gedeon Richter Plc. Budapest 1103, Hungary

## Abstract

Pain-related sensitization and synaptic plasticity in the central nucleus of the amygdala (CeA) depend on the endogenous activation of NMDA receptors and phosphorylation of the NR1 subunit through a PKA-dependent mechanism. Functional NMDA receptors are heteromeric assemblies of NR1 with NR2A-D or NR3A, B subunits. NMDA receptors composed of NR1 and NR2B subunits have been implicated in neuroplasticity and are present in the CeA. Here we used a selective NR2B antagonist (Ro-256981) to determine the contribution of NR2B-containing NMDA receptors to pain-related sensitization of CeA neurons. Extracellular single-unit recordings were made from CeA neurons in anesthetized adult male rats before and during the development of an acute arthritis. Arthritis was induced in one knee joint by intraarticular injections of kaolin and carrageenan. Brief (15 s) mechanical stimuli of innocuous (100–500 g/30 mm^2^) and noxious (1000–2000 g/30 mm^2^) intensity were applied to the knee and other parts of the body. In agreement with our previous studies, all CeA neurons developed increased background and evoked activity after arthritis induction. Ro-256981 (1, 10 and 100 μM; 15 min each) was administered into the CeA by microdialysis 5–6 h postinduction of arthritis. Ro-256981 concentration-dependently decreased evoked responses, but not background activity. This pattern of effect is different from that of an NMDA receptor antagonist (AP5) in our previous studies. AP5 (100 μM – 5 mM) inhibited background activity and evoked responses. The differential effects of AP5 and Ro-256981 may suggest that NMDA receptors containing the NR2B subunit are important but not sole contributors to pain-related changes of CeA neurons.

## Background

Functional NMDA receptors are heteromeric assemblies of NR1 subunits with NR2A-D or, less commonly, with NR3A, B subunits [1-7]. The NR1 subunit is essential for channel formation, Ca^2+ ^permeability and voltage-dependent Mg^2+ ^block, whereas NR2 subunits form the glutamate binding site and account for kinetic properties. NR2B-containing NMDA receptors have slower kinetics than those that include NR2A [8]. During development NR2B expression is gradually replaced with NR2A in most CNS neurons but not in the central nucleus of the amygdala (CeA) [9]. NR2B containing receptors have been implicated in synaptic plasticity, memory formation and pain modulation [see [10]].

NMDA receptor function in the CeA is increased in a model of arthritis pain [11,12]. NMDA receptor function can be modulated through phosphorylation of NR1 or NR2 subunits by various kinases, including PKA, PKC, ERK and tyrosine kinase [7,13-17]. Our previous studies showed that PKA-dependent phosphorylation of NR1 in the CeA is a key mechanism of increased responsiveness and synaptic plasticity in the arthritis pain model [11,12]. ERK activation also increases NMDA receptor function in the CeA, but PKC does not seem to be involved [18]. PKA activation appears to be downstream of CGRP1 receptors [19] and CRF1 receptors)[20,21]. NMDA receptors in the CeA do not contribute significantly to normal synaptic transmission and the processing of physiological nociceptive inputs [11,12]. The role of NR2B subunits in pain-related changes of CeA neurons is not known.

## Findings

Extracellular single-units were made from 8 neurons in the laterocapsular division of the CeA in 8 anesthetized male rats (250–350 g) as described in detail before)[21,22]. All experimental procedures were approved by the Institutional Animal Care and Use Committee (IACUC) at the University of Texas Medical Branch and conform to the guidelines of the International Association for the Study of Pain (IASP) and of the National Institutes of Health (NIH). Animals were mounted in a stereotaxic frame, paralyzed with pancuronium (induction: 0.3–0.5 mg, i.v.; maintenance: 0.3 mg/h, i.v.) and artificially ventilated (3–3.5 ml; 55–65 strokes/min). Constant levels of anesthesia were maintained by continuous i.v. infusion of pentobarbital (15 mg/kg per h). A small unilateral craniotomy was performed at the sutura frontoparietalis level for the recording of CeA neurons with glass-insulated carbon filament electrodes and for drug application by microdialysis (CMA11/Microdialysis Inc., North Chelmsford, MA; 8 kD cut-off, membrane diameter: 250 μm, membrane length: 1 mm). The following stereotaxic coordinates were used [23]: recording electrode, 2.1–2.8 mm caudal to bregma; 3.8–4.2 mm lateral to midline; depth 7–9 mm; microdialysis probe, 1.8 mm caudal to bregma; 4.0 mm lateral to midline; depth of tip 9.0 mm.

**Figure 1:**
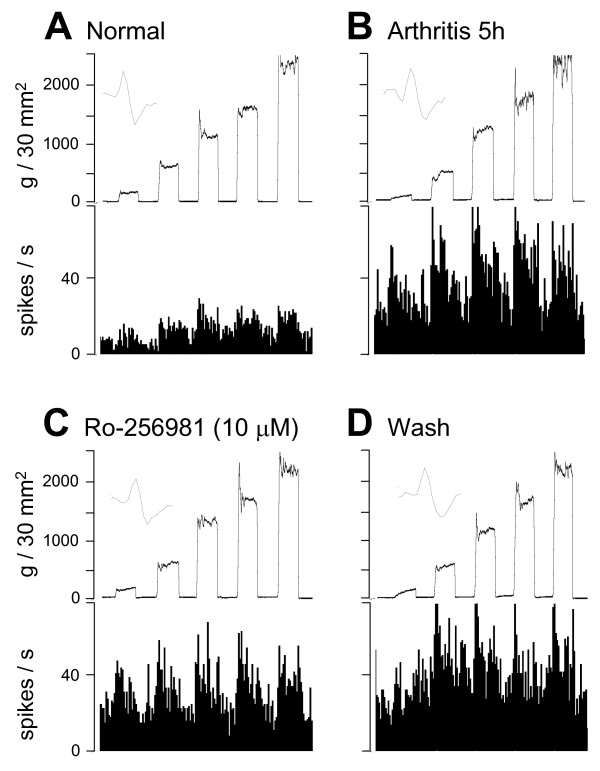
**Ro-256981 inhibits pain-related sensitization of an individual amygdala neuron**. Extracellular recordings of the responses (spikes/s) of one CeA neuron to brief (15s) graded mechanical stimulation (compression) of the knee. **(**A**) **In the control period before arthritis, the neuron responded more strongly to noxious than to innocuous stimuli. **(**B**) **The responses of the same neuron increased 5 h after induction of the arthritis in one knee. **(**C**) **Administration of Ro-256981 (10 μM, concentration in the microdialysis probe; 15 min) into the CeA inhibited the increased responses. **(**D**) **The inhibitory effect of Ro-256981 was largely reversible after wash out with ACSF for 30 min. **(**A-D**) **Top traces show recordings of the force (g/30 mm^2^) applied to the knee joint with a calibrated forceps. Peristimulus time histograms show number of action potentials (spikes) per second. Insets show that spike size and configuration remained constant throughout the experiment.

Background activity and responses evoked by brief (15 s) mechanical test stimuli of increasing intensities (100, 500, 1000, 1500 and 2000 g/30 mm^2 ^force, applied with a calibrated forceps at 15 s interstimulus intervals; see Fig. 1) were recorded before and after induction of a knee joint arthritis with intraarticular injections of kaolin and carrageenan [24]. Stimulus intensities of 100 and 500 g/30 mm^2 ^applied to the knee and other deep tissue are considered innocuous, whereas intensities of >1500 g/30 mm^2 ^are noxious because they evoke withdrawal reflexes and vocalizations in awake rats [25]. Background activity before stimulation was subtracted from the total response during stimulation to calculate the net response evoked by a particular stimulus.

**Figure 2:**
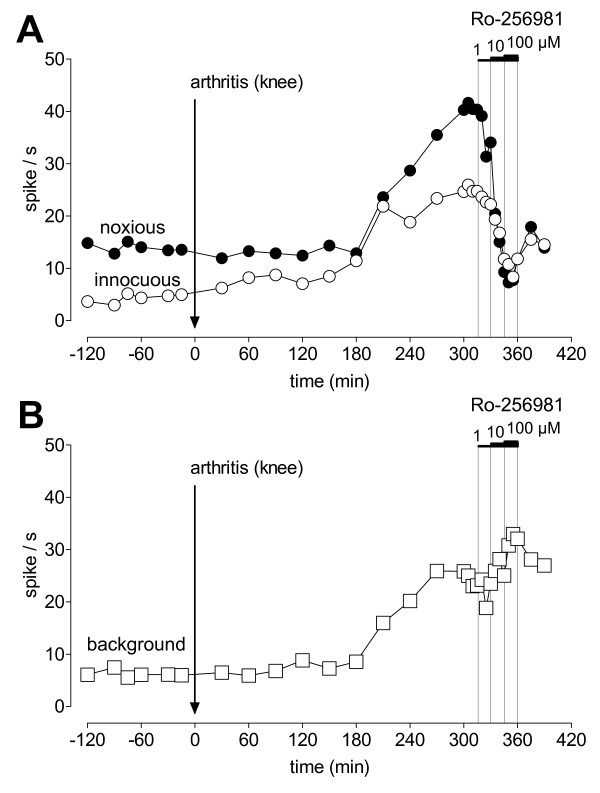
**Time course of the effects of arthritis and Ro-256981 in an individual amygdala neuron**. The responses of the CeA neuron to innocuous (500 g/30 mm^2^) and noxious (2000 g/30 mm^2^) stimulation of the knee (A) and background activity (B) increased after induction of the knee joint arthritis Administration of Ro-256981 (1, 10, and 100 μM; concentration in the microdialysis probe; 15 min) into the CeA reduced the enhanced responses (A) but not background activity (B) in a concentration-dependent fashion. Symbols show the mean activity during a 15 s period before stimulation (= background activity, B) and the difference between mean activity during and before 15 s stimuli (= net activity evoked by noxious or innocuous stimuli, A). Artificial cerebrospinal fluid (ACSF) was administered as a vehicle control before and after drug application.

In this study neurons were selected which had a receptive field in the knee and responded more strongly to noxious than innocuous stimuli. These so-called multireceptive (MR) neurons, and only MR neurons, become sensitized consistently in the arthritis pain model [11,19,22,26]. Figures 1 and 2 show individual examples. Background activity and evoked responses are enhanced after arthritis induction (Fig. 1B). Figure 2 illustrates the time course of pain-related changes. Responses to innocuous (500 g/30 mm^2^) and noxious (2000 g/30 mm^2^) stimulation (compression) of the knee (Fig. 2A) and background activity (Fig. 2B) increased within few hours after arthritis induction and reached a maximum plateau at 5 h postinduction.

A potent and selective NR2B receptor antagonist (Ro-256981) [27,28] was administered into the CeA through a microdialysis probe that had been inserted stereotaxically several hours before the experiment. Artificial cerebrospinal fluid (ACSF) was pumped through the fiber at a rate of 5 μl/min throughout the experiment to maintain stable conditions in the tissue. Ro-256981 (R-R*, S*)-α-(4-hydroxyphenyl)-β-methyl-4-(phenylmethyl)-1-piperidine propanol) was a gift from Gedeon Richter Ltd., Budapest, Hungary. Ro-256981 was dissolved in water to obtain stock solutions (10 mM). Stock solutions were diluted in ACSF to their final concentrations on the day of the experiment. Drug concentrations in the microdialysis fiber were 1.0, 10 and 100 μM, i.e., 100 times that predicted to be needed in the tissue based on data in the literature [27], because of the concentration gradient across the dialysis membrane [11,22,29]. Ro-256981 was only tested in the arthritis state because NMDA receptor antagonists have little or no effect on CeA neurons under normal conditions [11,12].

**Figure 3:**
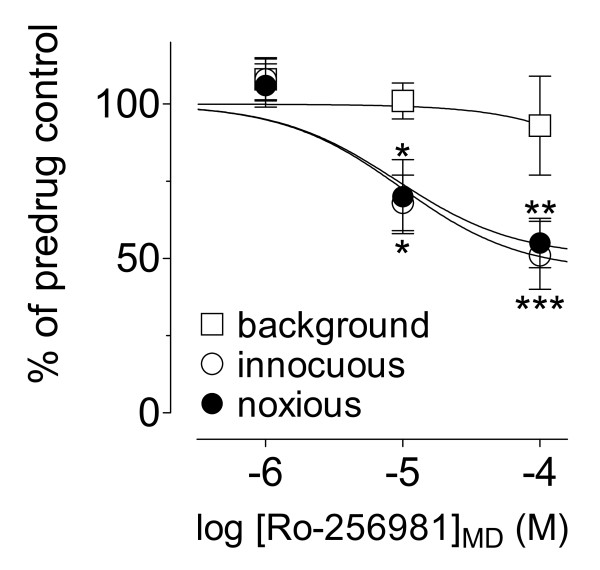
**Concentration-dependent inhibitory effects of Ro-256981 in the arthritis pain model**. Administration of Ro-256981 into the CeA 5–6 h postinduction of arthritis inhibited the responses of CeA neurons (n = 6–8 neurons for each concentration) to normally innocuous and noxious stimuli but not background activity. Numbers refer to the concentrations in the microdialysis probe (MD). Ro-256981 was administered into the CeA by microdialysis for 15 min. All averaged values are given as the mean ± SEM and are expressed as percent of predrug control values (in ACSF; set to 100%). The following formula was used for non-linear regression analysis to obtain concentration-response curves and calculate IC_50 _values: *y *= *A*+(*B*-*A*)/[1+(10^*C*^/10^*X*^)^*D*^], where *A *is the bottom plateau, *B *top plateau, *C *= log(IC_50_), and *D *is the slope coefficient (Prism 3.0, GraphPad Software). * P < 0.05, ** P < 0.01, *** P < 0.001, compared to predrug control values (Dunnett's multiple comparison test).

Administration of different concentrations (15 min each) of Ro-256981 into the CeA 5–6 h postinduction of arthritis decreased the evoked responses but not background activity (see individual examples in Figs. 1 and 2). The effects of Ro-256981 were concentration-dependent (Fig. 3; n = 8 neurons) and partially reversible after washout in ACSF for >30 min (Fig. 1D and Fig. 2A). Concentrations of 10 μM and 100 μM had significant effects (P < 0.05–0.001, Dunnett's multiple comparison test comparing the effects of individual concentrations of Ro-256981 to predrug control values). Non-linear regression analysis yielded apparent IC_50 _values of 7.9 μM and 9.3 μM for the inhibition of responses to normally innocuous and noxious stimuli, respectively.

Considering the concentration gradient across the dialysis membrane and diffusion in the tissue, these IC_50 _values are consistent with reported Kd values of 3 nM for the high-affinity binding Ro-256981 to rat forebrain membranes that contain different NMDA receptor subtypes [27]. They are also consistent with IC_50 _values for inhibitory effects of Ro-256981 on membrane currents evoked in oocytes coexpressing NR1C and NR2B (9 nM) or NR1F and NR2B (17 nM) and on NMDA-induced membrane currents in cultured rat cortical neurons expressing NR2B as the dominant NR2 subunit (15 nM) [27]. The selectivity of Ro-256981 for NR2B over NR2A is about 5000-fold [27].

Our data show that the endogenous activation of NR2B-containing receptors contributes critically to the increased evoked responses, but not background activity, of CeA neurons observed in the arthritis pain model. The diagnostic NMDA receptor antagonist AP5 inhibited evoked responses and background activity of CeA neurons in this pain model [11]. Furthermore, the effects of AP5 were greater on responses evoked by low- than high-intensity stimulation [11]. The differential effects of Ro-256981 and AP5 may suggest the involvement of other NMDA subtypes.

Accumulating evidence implicates NR2B-containing NMDA receptors in pain mechanisms and pain behavior. Systemic application of NR2B-selective antagonists such as CP-101,606 and Ro-256981 had antinociceptive effects in models of inflammatory and neuropathic pain [28,30,31]. Ro-256981 applied systemically or injected into the anterior cingulate cortex (ACC) inhibited allodynia-like behavior of mice in an inflammatory pain model [32]. Ro-256981 also decreased NMDA receptor-mediated synaptic currents in ACC neurons in slices from mice with hindpaw inflammation and this effect was greater than in slices from control mice [32]. Spinal administration of Ro-256981 decreased the responses of spinal dorsal horn neurons to electrical C-fiber stimulation and attenuated C-fiber evoked long-term potentiation [33]. Hindpaw inflammation increased the expression of NR2B, but not NR1 or NR2A, in the ACC [32] and increased tyrosine phosphorylation of NR2B, but not NR2A, in the spinal cord [34,35] but not ACC [32]. Overexpression of NR2B in the ACC and insular cortex of transgenic mice resulted in prolonged nocifensive behavior in the formalin pain test without altering acute nocifensive responses [36].

## Conclusion

This study shows for the first time that NR2B receptor activation in the amygdala (CeA) contributes to pain-related increases of responsiveness of CeA neurons in the arthritis pain model. Administration of a selective NR2B receptor antagonist (Ro-256981) into the CeA decreased evoked responses but not background activity of CeA 5–6 h postinduction of arthritis. The differential effects of Ro-256981 and the diagnostic NMDA receptor antagonist (AP5) measured in our previous study [11] suggest that other NMDA receptor subtypes may be involved as well. In agreement with previous studies in the spinal cord and anterior cingulate cortex (ACC), these results provide further evidence for an important role of NR2B in pain-related neuroplasticity.

## Competing interests

The authors declare that they have no competing financial interests. Horvath Csilla is employed by Gedeon Richter Ltd, Budapest, Hungary. The company provided the compound (Ro-256981) and partially supported the work as a reference study. The compound is commercially available from Sigma, St Louis, Missouri, USA, and was synthesized at Gedeon Richter Ltd. solely for research purposes. The company has no financial interest in this compound.

## Authors' contributions

GJ performed the electrophysiological recordings, analyzed data, and provided figures and the first draft of the manuscript. CH conceived and initiated the study and helped finalize the manuscript. CH and VN conceptualized the hypothesis. VN designed and supervised the experiments, directed the data analysis, and finalized the manuscript. All authors read and approved the manuscript.
